# Degradation pathway and microbial mechanism of high-concentration thiocyanate in gold mine tailings wastewater

**DOI:** 10.1039/d0ra03330h

**Published:** 2020-07-07

**Authors:** Lei Li, Fanyao Yue, Yancheng Li, Aijiang Yang, Jiang Li, Yang Lv, Xiong Zhong

**Affiliations:** College of Resources and Environmental Engineering, Guizhou University Guiyang Guizhou 550025 China ycli3@gzu.edu.cn; Guizhou Jinfeng Gold Mine Limited, Qianxinan Buyi and Miao Autonomous Prefecture Qianxinan Guizhou 550025 China; Guizhou Karst Environmental Ecosystems Observation and Research Station, Ministry of Education Guiyang Guizhou 550025 China

## Abstract

As one of the inorganic pollutants with the highest concentration in the waste water of gold tailings using biohydrometallurgy, thiocyanate (SCN^−^) was effectively degraded in this research adopting a two-stage activated sludge biological treatment, and the corresponding degradation pathway and microbial community characteristics in this process were also studied. The results showed that SCN^−^ at 1818.00 mg L^−1^ in the influent decreased to 0.68 mg L^−1^ after flowing through the two-stage activated sludge units. Raman spectroscopy was used to study the changes of relevant functional groups, finding that SCN^−^ was degraded in the COS pathway. Based on 16S rRNA high-throughput sequencing technology, the microbial diversity in this system was analyzed, and the results indicated that *Thiobacillus* played a major role in degrading SCN^−^, of which the abundance in these two activated sludge units was 32.05% and 20.37%, respectively. The results further revealed the biological removal mechanism of SCN^−^ in gold mine tailings wastewater.

## Abstract 2

Thiocyanate (SCN^−^) was degraded in COS pathway, and the removal rate was 99.94%. The *Thiobacillus* played an important role.

## Graphical abstract

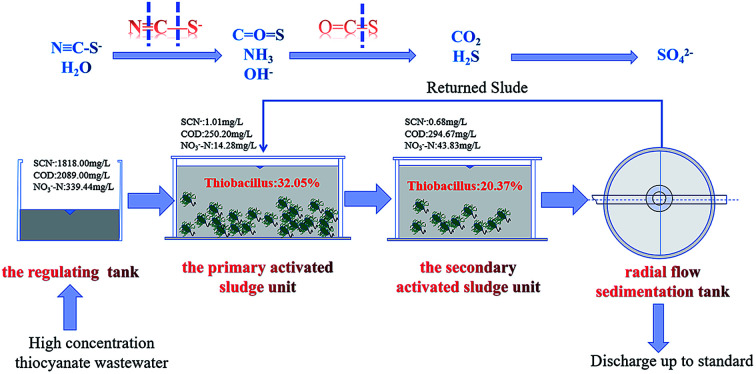

## 1 Introduction

China is rich in gold resources, most of which have been developed completely, with the rest hard to mine. In view of this, economical, efficient and environmentally-friendly biohydrometallurgy is explored across the world. However, when minerals are oxidized by microorganisms, sulfides or thiosulfates can react with cyanide to form a large number of sulfur cyanates (SCN^−^) containing carbon, nitrogen and sulfur, which are moderately toxic and chemically stable.^1^ Emission of SCN^−^ will do serious harm to the ecological environment.^2^ At present, there are few reports on the treatment of high-concentration SCN^−^ in the wastewater, and according to the existing data, the reducibility and adsorption capacity of SCN^−^ are stronger than that of CN^−^, and most degradation processes of CN^−^ such as alkaline chlorine oxidation,^3,4^ hydrogen peroxide oxidation,^5^ ozone oxidation,^6^ activated carbon adsorption oxidation,^7^ ion exchange, can be also used for the removal of SCN^−^ in wastewater. No matter which chemical or biological treatment is adopted, the conversion of SCN^−^ in aerobic conditions needs a lot of oxidants, which is costly and may produce intermediate product CN^−^, thus increasing the toxicity of wastewater.^7,8^ As an economic and environmental-friendly method, biohydrometallurgy is attracting more and more attention. Considering that SCN^−^ consists of S, C and N, which are all necessary elements for the growth of the living beings, it can provide a carbon source, nitrogen source and sulfur source for microorganisms under aerobic conditions, and generate such metabolites as SO_4_^2−^, CO_2_, and NH_4_^+^.^9^ Hence, high-load SCN^−^ in the wastewater can be biodegraded efficiently under the condition that dissolved oxygen and hydraulic retention time (HRT) are sufficient.

Biological treatment of SCN^−^ can be generally divided into two parts: the carbon removal unit to decompose and release CO_2_, and the nitrification and denitrification unit to remove NH_4_^+^–N from wastewater. Now, bacteria including *Arthrobacte*r, *Escherichia*, *Methylobacterium*,^9,10^*Bacillus*,^11^*Pseudomonas*, *Acinetobacter*,^12^*Thiobacillus*,^13^*Burkholderia*, *Chryseobacterium*,^14^*Klebsiella* and *Ralstonia*^15^ and others, been isolated and identified from many resources to degrade thiocyanate. Paruchuri *et al.* found that the mixed culture containing *Pseudomonas* and *Bacillus* could degrade SCN^−^ batch culture up to 1400 mg L^−1^ in 6 days.^16^ Chaudhari and Kodam isolated *Klebsiella pneumoniae* and *Ralisto*. sp co-cultures from thiocyanate-contaminated sites and found,^15^ the SCN^−^ degradation rate was 500 mg (L^−1^ d^−1^) at the concentration of 2500 mg L^−1^ thiocyanate at pH 6.0 and 37 °C. Different from physical and chemical treatment, biological treatment does not need a large number of oxidants and thus gradually becomes the research hotspot in respect of sulfur-containing cyanide wastewater treatment.

As a mature biological treatment process, activated sludge process plays an irreplaceable role in wastewater treatment. The large surface area and sugar layer of the activated sludge can quickly absorb pollutants in sewage, and effectively biodegrade pollutants relying on the relevant functional microorganisms. Hence, in this paper, a two-stage activated sludge biological treatment process is applied into the effective biodegradation of high-concentration thiocyanate (SCN^−^) in the gold mine tailings wastewater. Meanwhile, the functional groups in this system is analyzed to decode the degradation pathway of SCN^−^, and 16S rRNA high-throughput sequencing technology is also employed to analyze the microbial diversity in the system and further to explore the related biodegradation mechanism of SCN^−^.

## 2 Materials and methods

### 2.1 Water quality and method of the test

The tailing wastewater was selected from a gold mine in Guizhou Province, with the water quality shown as below: COD, 2089.00 mg L^−1^; SCN^−^, 1818.00 mg L^−1^; NH_4_^+^–N, 1.98 mg L^−1^; NO_2_^−^–N, 4.30 mg L^−1^; NO_3_^−^–N, 339.44 mg L^−1^; and TOC, 707.76 mg L^−1^, in a mode of “regulating tank + two-stage activated sludge units + radial flow sedimentation tank”. Influent flow reached 1000 m^3^ d^−1^ and the HRT of primary and secondary activated sludge units lasted 35.5 h and 10.5 h, respectively, with the MLSS being 4000 mg L^−1^, and the dissolved oxygen 2.5–3.5 mg L^−1^. Then, the mixture is separated in the radial flow sedimentation tank, and the separated sludge flowed back to the primary unit, with the reflux ratio reaching 200%. Sodium hydroxide solution was added to adjust pH value to 6–9 during operation. To ensure that a certain amount of organic matter can be provided for the growth and reproduction of microorganisms in the tanks, nutrition ratio of C : N : P should be maintained 100 : 5 : 1. A nutrition dosing system for phosphorus solution was established to keep the balance of C, N and P in the tanks. While the system became stable, wastewater quality in the regulating tank, the primary and the secondary activated sludge units was assayed, and the microorganism in these two units were also analyzed by 16S rRNA high-throughput sequencing.

### 2.2 Sample collection and pretreatment

Wastewater samples in the regulating tank was numbered ①, the primary activated sludge unit ②, and the secondary activated sludge unit ③. The sludge water mixture of the primary unit was marked A and the sludge water mixture of the secondary unit was marked B. Sample ①②③ of 1 L was refrigerated in a cleaned and cold plastic bucket, and stored at −4 °C in the laboratory, and Sample A and B of 1 L was collected and stored at −20 °C after centrifugation in the laboratory.

### 2.3 Water quality index analysis

The pH value was analyzed by HACH portable multi parameter digital analyzer, COD by rapid digestion spectrophotometry (HJ/T 399-2007), NO_3_^−^–N and NO_2_^−^–N by ultraviolet spectrophotometry (HJ/T 346–2007), NH_4_^+^–N by Nessler's reagent spectrophotometry (HJ 535-2009), TOC by NPOC (non purged organic carbon) in combustion oxidation non dispersive infrared absorption (HT 501-2009), SCN^−^ by Isonicotinic acid pyrazolone spectrophotometry (GB/T13897-92), and total mercury by cold atomic absorption spectrophotometry (HJ 597-2011).

### 2.4 Raman spectrum analysis

The water samples were frozen at −20 °C and then dried to powder. Then, a proper amount of powder was taken to the center of quartz slide for spectral integration using confocal micro Raman spectrometer (LRS-8, Shanghai, China), with excitation light source being 632.8 nm He–Ne laser, excitation wavelength 532.08 nm, and grating 1800 L mm^−1^; and this process lasted 10 s and was repeated 4 times, in order to obtain a relatively smooth spectrum ranging form 100 cm^−1^ to 4000 cm^−1^. In addition, every spectrum sample was repeatedly collected three times to improve the accuracy. All the spectra were collected in a dark room at the laboratory temperature (23 °C) and pressure (0.1 MPa). Original 8.0 software was used for data analysis.

### 2.5 16S rRNA high throughput sequencing analysis

Microorganisms in the two units were collected and frozen at −20 °C, and then sent to Shanghai Meiji for 16S rRNA high-throughput sequencing. An universal bacterial primer: 338F (5′-ACTCCTACGGGAGGCAGCAG-3′), was used to amplify V3–V5. Microbial DNA was extracted for PCR amplification and product purification pre-experiment. Passing the pre-experiment, the formal experiment could be initiated, using TransGen AP221-02: TransStart Fastpfu DNA Polymerase, 20 μL reaction system: 5 × FastPfu buffer 4 μL, 2.5 mmol L^−1^ dNTPs 2 μL, forward primer (5 μM) and reverse primer (5 μM) 0.8 μL, FastPfu polymer 0.4 μL, BSA 0.2 μL, and template DNA 10 ng, then, adding ddH2O to 20 μL, as well as ABI GeneAmp® 9700 PCR amplification instrument, with the reaction parameters: pre denaturation at 95 °C for 3 min, denaturation temperature at 95 °C for 30 s, annealing temperature at 55 °C for 30 s, extension temperature at 72 °C for 45 s, in 72 cycles, and extension at 72 °C for 10 min. After amplification, PCR product sample of 3 L were loaded and assayed by 2% agarose gel electrophoresis. After that, an Illumina platform library was constructed, and then the data obtained were analyzed for biological information, including OTU clustering, species annotation and classification, community composition and others.

## 3 Results and discussion

### 3.1 Degradation of SCN^−^ by activated sludge process

High-concentration SCN^−^ wastewater was treated by two-stage activated sludge biological treatment process, in the stable period, water quality of the influent and effluent was shown in Table 1. Among them, SCN^−^ decreased from 1818.00 mg L^−1^ to 1.01 mg L^−1^ and the removal rate reached 99.94% after the flowing through the primary unit, and then, the total removal rate of SCN^−^ increased to 99.96% after the secondary unit, which was consistent with the removal rate of COD (85.89%) and the reduction rate of TOC in water (93.89%). As a main source of COD in gold wastewater, decreases of SCN^−^ concentration can lead to COD decrease.

**Table 1:** Water quality features of influent and effluent in the gold mine tailing wastewater

Index	①	②	Removal efficiency 1	③	Removal efficiency 2	Total removal rate
pH	8.82	7.13	—	6.66	—	—
NH_4_^+^–N (mg L^−1^)	99.21	318	−220.53%	292	8.18%	−194.33%
NO_2_^−^–N (mg L^−1^)	4.3	0.275	93.6%	0.0174	93.67%	99.60%
NO_3_^−^–N (mg L^−1^)	339.44	14.28	95.79%	43.83	−67.42%	87.09%
COD (mg L^−1^)	2089	250.20	88.02%	294.67	−17.77%	85.89%
TOC (mg L^−1^)	707.76	41.918	94.08%	23.658	43.56%	93.89%
SCN^−^ (mg L^−1^)	1818	1.01	99.94%	0.68	32.67%	99.96%
Hg (ng mL^−1^)	47.79	46.74	2.20%	1.47	96.85%	96.92%

After the treatment in the primary unit, the removal rate of NO_2_^−^–N and NO_3_^−^–N in the wastewater reached 93.6% and 95.79%, respectively and only NH_4_^+^–N exhibited an upward trend from 99.21 mg L^−1^ to 318 mg L^−1^, suggesting NH_4_^+^–N was an intermediate product from SCN^−^ degradation which further led to the accumulation of NH_4_^+^–N due to a lack of electron acceptors in the system. Theoretically, 100 mg SCN^−^ can release 24 mg NH_4_^+^–N, about 10% of which can be transformed into biomass by microorganism as nitrogen source, and the rest enter into the water body in a form of NH_4_^+^, resulting in the increase of NH_4_^+^–N concentration in water.^12^ On the other hand, Huang *et al.* found that the nitrification of NH_4_^+^-N was inhibited due to the existence of SCN^−^.^14^

Formula (1): SCN^−^ conversion path.

1 SCN^−^ + H_2_O → S_2_^−^ + NH_4_^+^ + CO_2_

Then, after flowing through the secondary unit, NH_4_^+^–N in the wastewater decreased by 26 mg L^−1^, but NO_3_^−^–N increased by 29.55 mg L^−1^, showing nitrification occurred in this secondary unit. Moreover, it was found that mercury in this unit reduced from 47.79 ng mL^−1^ to 1.47 ng mL^−1^ with the removal efficiency high up to 96.92%, suggesting this system had a favorable condition to remove mercury.

### 3.2 Raman analysis of SCN^−^ degradation pathway

The results showed that there were two pathways to degrade SCN^−^. One was COS pathway. Under the action of thiocyanate hydrolase, the nitrogen carbon bond in SCN^−^ hydrolyzed to COS and NH_3_, where the C–S bond was broken into CO_2_ and H_2_S, and H_2_S oxidized to SO_4_^2−^. The other was CNO pathway. The sulfur carbon bond in SCN^−^ was broken and hydrolyzed to cyanate (CNO^−^) and HS^−^, where CNO^−^ was hydrolyzed into NH_3_ and CO_2_ by cyanate hydrolysis enzyme, and S^2−^ was further oxidized to sulfide and SO_4_^2−^.^9^

The COS pathway:^15^

2 N

<svg xmlns="http://www.w3.org/2000/svg" version="1.0" width="23.636364pt" height="16.000000pt" viewBox="0 0 23.636364 16.000000" preserveAspectRatio="xMidYMid meet"><metadata>
Created by potrace 1.16, written by Peter Selinger 2001-2019
</metadata><g transform="translate(1.000000,15.000000) scale(0.015909,-0.015909)" fill="currentColor" stroke="none"><path d="M80 600 l0 -40 600 0 600 0 0 40 0 40 -600 0 -600 0 0 -40z M80 440 l0 -40 600 0 600 0 0 40 0 40 -600 0 -600 0 0 -40z M80 280 l0 -40 600 0 600 0 0 40 0 40 -600 0 -600 0 0 -40z"/></g></svg>

C–S^−^ + 2H_2_O → O

<svg xmlns="http://www.w3.org/2000/svg" version="1.0" width="13.200000pt" height="16.000000pt" viewBox="0 0 13.200000 16.000000" preserveAspectRatio="xMidYMid meet"><metadata>
Created by potrace 1.16, written by Peter Selinger 2001-2019
</metadata><g transform="translate(1.000000,15.000000) scale(0.017500,-0.017500)" fill="currentColor" stroke="none"><path d="M0 440 l0 -40 320 0 320 0 0 40 0 40 -320 0 -320 0 0 -40z M0 280 l0 -40 320 0 320 0 0 40 0 40 -320 0 -320 0 0 -40z"/></g></svg>

CS + NH_3_ + OH^−^

3 OCS + H_2_O → H_2_S + CO_2_

**Fig. 1:**
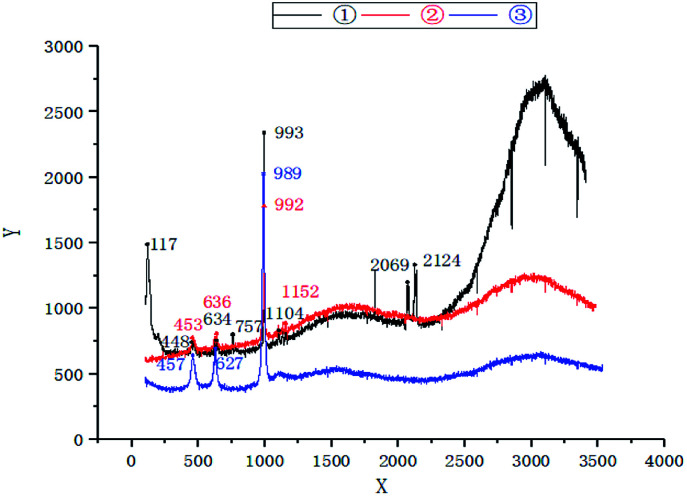
Raman spectrum analysis of water quality—①: regulating tank; ②: primary activated sludge unit; ③: secondary activated sludge unit.

It can be seen from the analysis of three groups of data by comparing the Raman spectrogram (Fig. 1) and Raman spectrogram analysis (Table 2) of water quality, there were two vibrations in the absorption band of sulfur carbon C–S located at 730–600 cm^−1^ in the regulating tank, specifically speaking, 634 cm^−1^ and 757 cm^−1^, respectively. In the primary unit, there was only one vibration at 636 cm^−1^, and in the secondary unit, there was only one vibration at 627 cm^−1^, with the vibration frequency less than that in the regulating tank and the primary unit. At the same time, the C≡N stretching vibration frequency of M–S–C≡N, a metal thiocyanate salt located at 2160–2040 cm^−1^, also appears in the water of the regulating tank.^20,21^ Tian *et al.* reported that the S-and N-combined thiocyanate showed C–N stretching vibration in the range of 2050–2165 cm^−1^, that is to say, this peak belongs to C≡N stretching vibration, and the absorption band in the secondary activated sludge unit is the weakest, indicating that the C–S bond and C≡N bond fracture of SCN^−^ (electronic formula is N≡C–S^−^) mainly occur in the primary activated sludge unit. The C=S stretching vibration peak at 1200–1020 cm^−1^ only appeared in the regulating tank and the primary effluent, and disappeared in the secondary effluent, indicating that the C=S bond fracture of O=C=S mainly occurred in the secondary unit.^22^ In Minogue's report, the Raman spectral frequencies of 981 cm^−1^ and 451 cm^−1^ belong to the vibration of SO_4_^2−^. The vibration frequency (about 460 cm^−1^) caused by the symmetric angle change of SO_4_^2−^ from the regulating tank to the primary effluent changed slightly, but then weakened to the lowest extent in the secondary effluent; however, the S–O symmetric stretching vibration peak (about 980 cm^−1^) of SO_4_^2−^ was very strong at the beginning, and then weakened gradually from the regulating tank to the primary effluent and then to the secondary effluent, indicating that H_2_S can be further oxidized into SO_4_^2−^ both in the primary and secondary units. Accordingly, Raman spectrum analysis indicated that biodegradation of SCN^−^ belonged to COS pathway, where the N–C bond and S–C bond in SCN^−^ was hydrolyzed into COS and NH_3_ in the primary unit; then the C–S bond in COS was broken into CO_2_ and H_2_S mainly in the secondary unit; and finally, H_2_S was further oxidized into SO_4_^2−^.

**Table 2:** Result of Raman spectroscopic analysis

Vibration mode	Vibration frequency	References
The symmetric angle of SO_4_^2−^	460 cm^−1^	17 and 18
Sulfur carbon C–S	730–600 cm^−1^	19
SO_4_^2−^ symmetric expansion	1000–950 cm^−1^ (about 980 cm^−1^)	17 and 18
C=S expansion vibration	1200–1020 cm^−1^	20
M–S–C≡N expansion	2160–2040 cm^−1^	20 and 21

### 3.3 Analysis of microbial community structure

Biofilms in the primary and secondary units were sampled for 16S rRNA high-throughput sequencing to analyze the bacteria community structure that degraded SCN^−^, and OTU clustering was carried out for non-repetitive sequences (excluding single sequences) according to 97% similarity by calculating common diversity indexes (Table 3) including richness index (Chao/ACE index), coverage index, Shannon index and Simpson index.

**Table 3:** Diversity indexes

Sample	Sequences	OTUs	Shannon	ACE richness	Chao richness	Courage
A	38 146	154	2.65	163.38	159.22	0.9996
B	35 886	151	2.81	160.50	158.16	0.9995

**Fig. 2:**
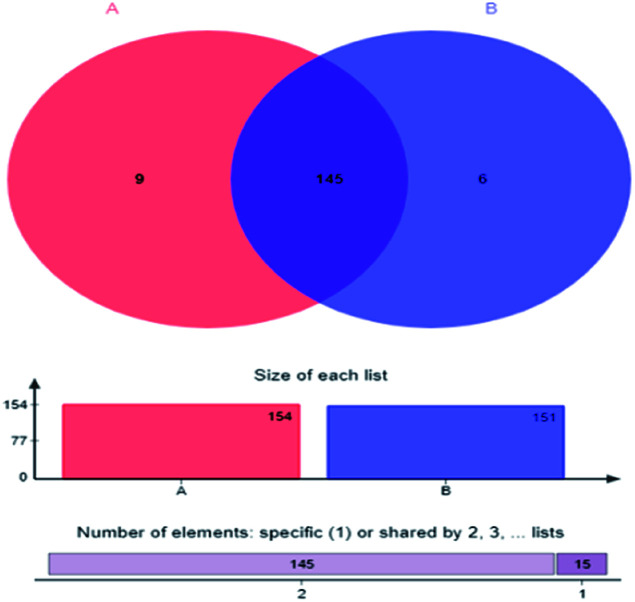
Venn diagram of microbial community—A: primary activated sludge unit; B: secondary activated sludge unit.

Couerage index refers to the sequencing depth and coverage rate of the samples. Coverage rates of sample A and B was 0.9996 and 0.9995, respectively, indicating that most of the bacterial populations were detected. The small difference among Shannon index, Simpson index, ACE index and Chao index of sample A and B, as well as the Venn diagram of sample A and B microbial communities (Fig. 2) where the two sludge samples shared 90.63% of the total OTUs, proved similar microbial community diversity between them.

**Fig. 3:**
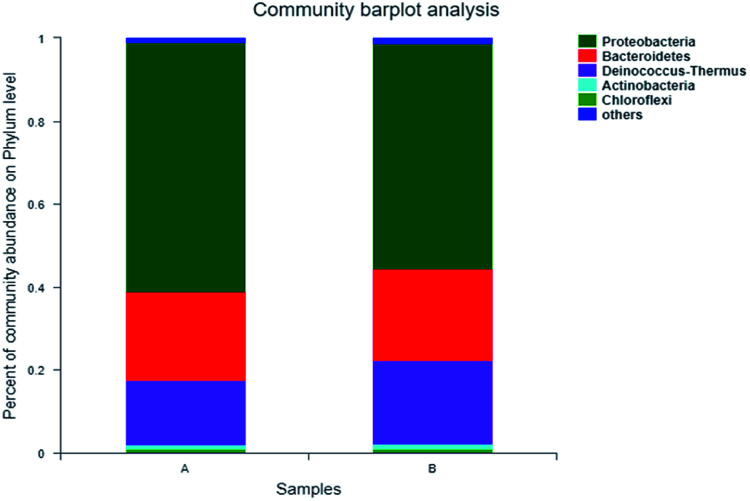
Microbial classification at a Phylum level—A: primary activated sludge unit; B: secondary activated sludge unit.

Further taxonomic analysis of OTU representative sequences found that over 95% of the microbial communities of A and B in the entire reaction were composed of three phylum-level bacterial communities (Fig. 3), referring to Proteobacteria, Bacteroidetes and Deinococcus Thermus, respectively, of which Proteobacteria was the dominant with the respective abundance reaching 60.6% and 54.9%. Bacteroidetes was the second dominant, accounting for 21.7% and 22.5%, respectively. Manz *et al.* found that Proteobacteria possessed the capacity of water treatment.^23^ McLellan *et al.* also found that Bacteroidetes is the dominant bacteria in the influent of sewage treatment plant.^24^

**Fig. 4:**
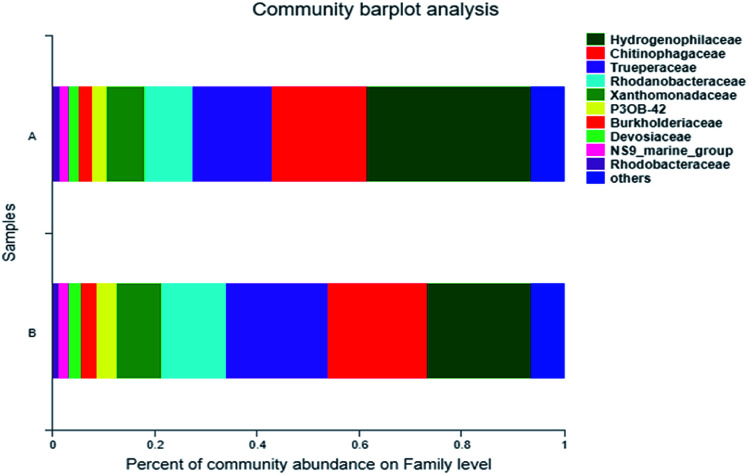
Microbial classification at a Family level—A: primary activated sludge unit; B: secondary activated sludge unit.

The dominant family-level bacteria of the two samples were Hydrophillaceae, Chitinophagaceae and Trueperaceae, with the similar relative abundance (Fig. 4). Relative abundances of Hydrogenopholaceae in the sample A and B were 32.05% and 20.37%, respectively. *Thiobacillus*, which has been reported to be capable of degrading SCN^−^, belongs to this family, and it is a special autotrophic bacterium which can utilize oxygen, nitrate and nitrite as electron acceptors to oxidize sulfide, thiosulfate and sulfur, so as to obtain energy.^25,26^ The respective relative abundance of Chitinophagaceae was 18.5% and 19.34%, and Trueperaceae was 15.58% and 19.96%. In addition, Burkholderiaceae, another one that can degrade SCN^−^, accounted for 2.65% and 2.9%, respectively, in these two samples. As a degrading microorganism that can grow and metabolize with thiocyanate as the only carbon source. *Burkholderia* sp. can completely degrade SCN^−^ of about 500 mg L^−1^ in 90 h.^14^ It is reported that Burkholderiaceae can also degrade hydrocarbons, phenol and PAHs (pyrene) in coking wastewater.^27,28^

**Fig. 5:**
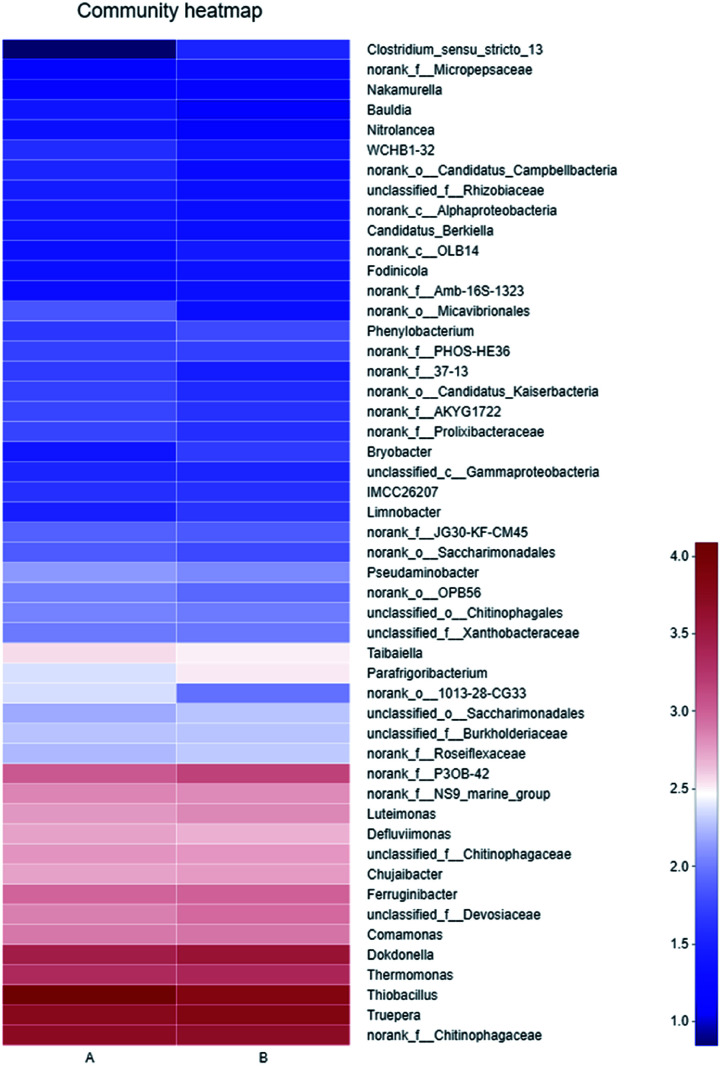
Heatmap of microbial community (A: primary activated sludge unit; B: secondary activated sludge unit).

As shown in Fig. 5, the dominant genera-level bacteria of the two samples were *Thiobacillus* (32.05% and 20.37%), *Truepera* (15.58% and 19.96%), *Chitinophagaceae* (13.45% and 14.02%), and *Dokdonella* (7.95% and 10.94%). Oxidation–reduction sulfide or elemental sulfur of *Thiobacillus* was sulfuric acid, and as a specific autotrophic bacterum using chemical energy, *Thiobacillus* could effectively degrade SCN^−^, during which SCN^−^ would generate metabolites such as NH_3_, NO_2_^−^ and S_2_^−^.^29^

The results showed that thiocyanate can be degraded in two pathways: *Thiobacillus*, a SCN^−^ degrading bacterium isolated from coking wastewater treatment plant, belonged to COS degradation pathway,^30^ while *Pseudomonas Putida* and *Pseudomonas* stutzeri, separated from gold smelting wastewater polluted soil, belonged to CNO degradation pathway.^31^*Thiobacillus* was the dominant strain in this experiment, suggesting the degradation of SCN^−^ belonged to COS degradation pathway,^30^ consistent with the afore results of Raman spectrum analysis.

Moreover, results of this experiment can reaffirm some of other researches. For example, Kelly *et al.* put forward that COS can generate CO_2_ and H_2_S through C–S bond fracture,^32^ and H_2_S can further oxidize to generate SO_4_^2−^, where the resulting energy can also facilitate the growth of microorganisms. Stratford *et al.* and Arakawa *et al.* extracted thiocyanate hydrolase from the isolated strain and identified that thiocyanate hydrolase was an inducible enzyme.^33,34^ Huddy *et al.* proved that *Thiobacillus* was the dominant bacteria to degrade SCN^−^ in ASTER™ biological treatment system using gene clone library analysis.^35^ In addition, the anammox bacteria *Candidatus Campbell* bacteria was detected, but its abundance was very small, about 0.17%, so it exerted a poor effect on the removal of NH_4_^+^–N.

The sequencing results showed that the nitrifying bacteria and nitrobacteria were not the dominant, and were excluded in the community Heatmap diagram (Fig. 5). What's more, results in Table 1 also verified that the ammonia nitrogen in this system did not decline. The reason might be that *Thiobacillus* became the dominant bacteria, and oxidized sulfur to sulfate, causing the pH value of the system decreased. However, nitrifying bacteria prefer a relatively weak alkaline environment. Kim *et al.* also studied that nitrifying bacteria in aerobic sludge cannot be detected due to the inhibition of toxic compounds.^36^ Hence, nitrifying bacteria were not dominant in this experimental condition.

## 4 Conclusions

In this research, high-concentration SCN^−^ gold tailing wastewater was treated in a two-stage serial activated sludge process, where SCN^−^ of 1818 mg L^−1^ was reduced to 0.68 mg L^−1^, with the degradation efficiency high up to 99.96%. In addition, the removal efficiency of COD, TN and Hg reached 85.89%, 85.51% and 96.92%, respectively. Meanwhile, according to Raman spectrum analysis of the functional groups' changes, SCN^−^ was transformed and degraded in the same COS pathway as thiocyanate was biodegraded. Analysis of 16S rRNA high-throughput sequencing identified. *Thiobacillus* was the dominant bacteria to degrade SCN^−^, with the abundance of 32.05% and 20.37% respectively in the two-stage activated sludge units.

## Conflicts of interest

The authors declare that there are no conflicts to declare.

## Supplementary Material
